# Loss of Eicosapentaenoic Acid (EPA) after Retort Sterilization of the EPA-BCAA Fortified Complete Nutrition Drink

**DOI:** 10.3390/foods11142023

**Published:** 2022-07-08

**Authors:** Watcharapol Khoonin, Prapimporn Chattranukulchai Shantavasinkul, Chalat Santivarangkna, Dunyaporn Trachootham

**Affiliations:** 1 Doctor of Philosophy Program in Nutrition, Faculty of Medicine, Ramathibodi Hospital, and Institute of Nutrition, Mahidol University, Nakhon Pathom 73170, Thailand; watcharapol.k@gmail.com; 2 Division of Nutrition and Biochemical Medicine, Department of Medicine, Faculty of Medicine Ramathibodi Hospital, Mahidol University, Bangkok 10400, Thailand; sprapimporn@gmail.com; 3 Graduate Program in Nutrition, Faculty of Medicine Ramathibodi Hospital, Mahidol University, Bangkok 10400, Thailand; 4 Institute of Nutrition, Mahidol University, Nakhon Pathom 73170, Thailand; chalat.san@mahidol.ac.th

**Keywords:** retort processing, complete nutrition drink, functional ingredients, fortification, EPA, BCAA, sensory property, personalized nutrition

## Abstract

Retort sterilization is cost-effective for small-scale production of specialized nutrition products. However, the sensory properties and stability of active ingredients after sterilization remain undetermined. This study aimed to investigate the effect of retort on the existence of functional compounds and the sensory satisfaction of a fortified complete nutrition formula with branched-chain amino acids (BCAAs), and fish oil providing eicosapentaenoic acid (EPA). Changes in EPA and BCAA contents after retort were determined by using LC-MS/MS. Nutrient values, osmolality, rheology and sensory acceptance of the processed fortified and control formulas were compared. After retort, the fortified formula had an increase in all types of BCAAs but 30% loss of EPA (*p* = 0.001). The fortified formula had slightly higher protein and fiber contents, along with increased osmolality. It had higher viscosity and shear stress, but similar IDDSI level at 0. Among flavors tested, the fortified formula with Japanese rice flavor received the highest satisfaction scores with over 80% sensory acceptance. In conclusion, retort sterilization preserved BCAAs of the functional drink, but the addition of 30% fish oil was required to compensate for the EPA loss. The sterilized fortified formula with Japanese rice flavor was sensory acceptable.

## 1. Introduction

Nutrition intervention is intended to fulfill individual nutrient requirements. When regular meals cannot meet the energy and nutritional needs of the elderly, the liquid complete nutrition formula is frequently selected to complete the personal nutrition goal [1]. The nutritional formulas have been created over the past several decades with a variety of intended uses to promote clinical outcomes. Numerous new formulas use a variety of terms such as nutrition drink, and enteral formula for tube feeding [2]. While standard formulations were intended to achieve the basic macro- and micronutrient requirements of individuals, specialty formulas have been developed for special needs such as immune-enhancing formulas containing arginine, glutamine, nucleotides, and ω-3 fatty acids [3]. The products must meet the safety standard and be shelf-stable at room temperature, meet optimum nutrient requirements, possess proper osmolality, and include adequate functional compounds [4]. Commercially available complete nutrition products are mostly sold in powder form. However, improper preparation of liquid food from powder results in adverse events, including blockage of tube feeding, poor absorption, and inadequate nutrient intake [5]. Therefore, a ready-to-eat complete nutrition formula is needed. 

Considering safety as a critical property of food for special medical purposes, thermal processing is still the most effective means to appropriately sterilize foodstuffs [6]. Ultra-high temperature (UHT) processing with aseptic filling is considered one of the best ways to sterilize food while preserving nutrients and sensory properties [7,8]. However, significant loss of vitamin C and B can still be found after UHT processing [9]. Furthermore, the technique requires a large volume of production due to the significant loss of raw materials during processing [8]. Thus, UHT-aseptic filling is suitable for manufacturing large batches of standard formulas but not small batches of personalized or specialized products. Among thermal processing methods, retort sterilization is considered the most cost-effective strategy for small-scale production of low acidic food [10]. Previous studies showed that retort processing of food products could result in undesirable sensorial and nutritional alterations and the loss of heat-sensitive nutrients such as vitamin C and polyunsaturated fatty acids [11]. Although the nutrient loss in conventional complete nutrition formulas after sterilization is well recognized, the amounts of functional compounds remaining in a special formula thereafter are unclear. 

To close the above gap of knowledge, this study aimed to investigate the effect of retort on the existence of functional compounds and the sensory satisfaction with a fortified complete nutrition formula containing branched-chain amino acids (BCAAs) and fish oil providing eicosapentaenoic acid (EPA). Recently, we developed a specialized nutritious liquid containing the functional compounds EPA and BCAA with the aim of preventing sarcopenia. Sarcopenia is a progressive loss of muscle caused by muscle degradation associated with malnutrition, aging, and chronic inflammation [12,13]. The functional formula is intended to provide an anti-inflammatory effect from EPA [14] and promote muscle synthesis by supplying BCAA [15]. To create the fortified formula, EPA and BCAA were added into a conventional liquid complete nutrition formula. Flavoring agents were added to mask the fishy odor of fish oil, the bitterness of amino acids, and the soy-like taste of soy protein in the nutrition formula. In this study, the sterilization process was selected to achieve the lethality value (F0) and ensure sterility. The impact of retort processing on added nutritional value was evaluated. Changes in osmolality and sensory satisfaction of the processed products were compared with those of the control formula.

## 2. Materials and Methods

### 2.1. Formula Preparation

For both control and fortified formulas, a conventional formula (Blendera-MF, Thai Otsuka Pharmaceutical Co., Ltd., Samut Sakhon, Thailand) was mixed with water at a ratio of 1 kcal per milliliter of water, using an automatic mixer at the rate of 350 rpm at 65 °C for 15 min. Only for the fortified formula, the fish oil containing 1.1 g of EPA per serving (PronovaPure^®^, BASF Pharma, Florham Park, NJ, USA) and BCAAs including 1.66 g of leucine, 0.42 g of isoleucine, and 0.42 g of valine per serving (Ajinomoto Co., (Thailand) Ltd., Bangkok, Thailand), were added and mixed into the control formula liquid. A variety of heat-stable flavoring agents were added, and the sensory acceptance of the various flavored fortified products was compared. The ingredients of the control and fortified formulas are shown in Table 1.

### 2.2. Sterilization

For one package, 250 mL of the liquid mixture was filled into a laminated retort pouch and sealed tightly. The horizontal stationary retorting system (Km Grand Pack Co., Ltd., Samut Prakan, Thailand) at the pilot plant of the Institute of Nutrition was used for sterilization. All of the packages were heated in a pressurized retorting system at the specified temperature–time profiles (116 °C− 25 min), which yielded a lethality (F0) value of 6.3 for the control formula and of 5.08 for the fortified formula. The graphical plot of the heat penetration study is shown in Figure S1. The heat profile was determined with the TrackSense ^®^ Pro Micro Wireless, Ellab AS, Hillerød, Denmark thermocouple sensor (Ellab AS, Hillerød, Denmark).

We produced 10 L of each formula for batch processing. In this study, after the processing condition for F0 was found, we manufactured one batch of each formula to obtain the physical, chemical, sensory, and microbiological properties.

### 2.3. Physicochemical Properties

Proximate composition was analyzed according to the AOAC Official Methods. Crude protein was assessed by the Kjeldahl method using a conversion factor of 6.25 (AOAC 991.20). Crude fat content was determined by solvent extraction (acid hydrolysis methods) using the Soxhlet apparatus (AOAC 922.06). Moisture content was analyzed by the vacuum drying method (AOAC 925.45). Ash content was analyzed by incinerating the sample in a muffle furnace (AOAC 945.46). Carbohydrate was calculated by deduction of crude fat protein, fat, moisture, and ash from a total of 100%. Total dietary fiber was determined by the enzymatic-gravimetric method (AOAC 985.29). The energy was calculated by multiplying the weight of protein, carbohydrate, and fat content with the conversion factor values of 4, 4, and 9, respectively. Microbiological analysis was performed by an ISO/IEC 17025 certified laboratory. The tests followed AOAC methods for low-acid canned food. Osmolality was measured using a single-sample osmometer (Advance Instrument LLC, Norwood, MA, USA). The color parameters of finished products were evaluated with a colorimeter (Hunterlab model ColorFlex EZ, Hunter Associates Laboratory, Inc., Reston, VA, USA). pH was measured by pH meter (Metler Toledo, Columbus, OH, USA). The rheological properties of the control and fortified formulas were measured with a Thermo Electron Corporation HAAKE MARS modular advanced rheometer system with a cup and rotor viscometer (cup CCB25 DIN/SS and rotor CC25 DIN/Ti). The temperature was controlled by a thermostatic bath set at 25 °C. The results were processed with the HAAKE RheoWIN Job Manager and the HAAKE RheoWIN Data Manager software. Rheology curves were provided in a shear rate interval between 0.0001 and 1000 s^−1^, and data points were collected (Thermo Electron Corporation, Karlsruhe, Germany). Texture categorization was evaluated by using the IDDSI flow test (https://iddsi.org/Testing-Methods, accessed on 1 June 2022). The complete nutrition liquid was filled in a 10 mL syringe. The liquid was allowed to flow through the syringe for 10 s. Then, the remaining amount of liquid in the syringe was measured. The liquid would be categorized as IDDSI level 0, 1, 2, 3, or 4 when less than 1 mL, 1 to less than 4 mL, 4 to less than 8 mL, 8 to less than 10 mL, and 10 mL remained, respectively.

### 2.4. Analysis of EPA

One mL of sample was collected from the conventional and fortified complete nutrition formulas before and after the retort sterilization. For EPA analysis, the lipid extraction method with hexane/isopropanol previously described in the analysis of EPA in salmon and plasma was modified to use in this study [16,17]. The processed liquid food was extracted with 3:2 volume by volume of hexane:isopropanol at 1:10 volume by volume of sample to solvent. After mixing, the mixture was incubated at −20 °C for 10 min, followed by centrifugation at 14,000× *g* at 4 °C for 5 min. Then, the hexane extract layer was collected and dried to remove solvents using a SpeedVac concentrator (CentriVap Benchtop Vacuum Concentrators, Labconco, Kansas City, MO, USA) at room temperature for 15 min. One mL of 80% methanol was added to the sample. Nine hundred microliters of the extract underwent alkaline hydrolysis by the addition of 100 µL of 0.3 M KOH in 80% methanol, followed by incubation at 80 °C for 30 min. After the sample was cooled down, 10 µL of formic acid was added to neutralize the pH. One milliliter of hexane was added for the extraction of fatty acids, mixed for 5 min, and centrifuged at 1000× *g*. The hexane layer was collected and evaporated with a SpeedVac concentrator to remove the solvent. Then, the sample was reconstituted with 1 mL of 80% methanol and filtered through a 0.2 µm Nylon filter prior to the liquid chromatography-tandem mass spectrometry (LC-MS/MS) analysis. For the calibration curve generation, 0, 125, 250, 500, 750, and 1000 ng/mL of EPA in 80% methanol were used. An internal standard, EPA-d5 (MaxSpec, Cayman Chemical, Ann Arbor, MI, USA), was added equally to all samples and standard solutions at the beginning of sample preparation.

Liquid chromatography was performed using an Ultimate 3000 Ultra High-performance liquid chromatography (UHPLC, Thermo Scientific, Waltham, MA, USA) equipped with a Hypersil GOLD™ C18 column (100 × 2.1 mm, particle size 1.9 μm) at 50 °C. The mobile phases A and B were acetonitrile and 5 mM ammonium acetate in water, respectively. The flow rate was 0.6 mL/min with a run time of 10 min. The gradient run included at 0–6.5 min: 40% B, at 6.5–9 min: 2% B, and at 9–10 min: 40% B. The injection volume was 5 μL. The retention times of EPA and EPA-d3 were equal at 1.7 min. Mass spectrometry (MS) was conducted using a TSQ Quantis triple quadrupole mass spectrometer (Thermo Scientific, USA). The MS condition was negative ion electrospray ionization (−ESI) with spraying voltage at 3500 V under N2 Sheath. Sheath and auxiliary gases were 30 and 15 arbitrary units (Arb). Ion transfer tube (ITT) and vaporizing temperatures were 325 °C and 350 °C, respectively. The mass spectrometer analysis was performed in selected reaction monitoring (SRM) mode for simultaneous analysis of multiple masses (Table S1). The mass to charge ratios (*m/z*) of EPA precursor and quantified product masses were 301 and 257, respectively. A collision energy of 12 V was used for the transition. The confirmation product mass for EPA was *m/z* 203.3 with a collision energy of 13 V for the transition. The mass to charge ratios (*m/z*) of EPA-d5 precursor and quantified product masses were 306.3 and 262.3, respectively. A collision energy of 12 V was used for the transition.

To optimize analytical conditions determining BCAA and EPA contents in the formulas, the LC conditions and ESI sensitivity that affect the sensitivity and separation of each compound were retrieved from previous studies on a blood sample [17,18]. Internal calibration of each compound was performed by using the isotopic forms of the respective standard as the internal standard. 

### 2.5. Analysis of BCAAs

Acid hydrolysis was performed as previously described to release all available amino acids in the complete nutrition liquid. The protein was hydrolyzed by using 6 N HCl solution at 120 °C for 2 h [19]. The dried sample was then reconstituted with 0.02 M HCl and centrifuged at 13,000 rpm for 5 min [20]. The supernatant was collected, and the final volume of 5 mL was adjusted with water. Then, the sample was filtered through a 0.2 µm Nylon filter and kept in a glass vial for further analysis. The metabolomics amino acid mixtures unlabeled standard (MSK-A2-US-1.2: Cambridge Isotope Laboratories, Inc., Tewksbury, MA, USA) was used for the preparation of standard solutions of valine, isoleucine, and leucine. Internal standards including 13C/15N-valine, 13C/15N-isoleucine, and 13C/15N-leucine were supplied by stable isotope-labeled metabolomics amino acid mixtures (MSK-A2-1.2: Cambridge Isotope Laboratories, Inc., Tewksbury, MA, USA). 

Liquid chromatography was performed using an Ultimate 3000 Ultra High-performance liquid chromatography (UHPLC; Thermo Scientific, Waltham, MA, USA) equipped with a Hypersil GOLD™ C18 column (100 × 2.1 mm, particle size 1.9 μm) at 30 °C. The mobile phase was 5:95 of methanol: 20 mM ammonium formate in water. The flow rate was 0.3 mL/min with a run time of 5 min. The injection volume was 5 μL. The retention times of valine, 13C/15N-valine, isoleucine, 13C/15N-isoleucine, leucine, and 13C/15N-leucine were 1.21, 1.19, 1.81, 1.85, 1.98, and 2.01 min, respectively. Mass spectrometry (MS) was conducted using a TSQ Quantis triple quadrupole mass spectrometer (Thermo Scientific, Waltham, MA, USA). The MS condition was composed of positive ion electrospray ionization (+ESI) combined with spraying voltage at 3500 V under N2 Sheath. Sheath, auxiliary and sweep gases were 50, 10, and 1 Arbs. Ion transfer tube (ITT) and vaporizing temperatures were 325 °C and 350 °C, respectively. The mass spectrometry analysis was performed in selected reaction monitoring (SRM) mode for simultaneous analysis of multiple masses, as shown in Table S1. The mass to charge ratios (*m/z*) of valine precursor and quantified product masses were 118 and 72, respectively. The collision energy of 10 V was used for the transition. The confirmation product mass for valine was *m/z* 55 with a collision energy of 21 V for the transition. The mass to charge ratios (*m/z*) of 13C/ 15N-valine precursor and quantified product masses were 306.3 and 262.3, respectively. A collision energy of 11 V was used for the transition. The mass to charge ratios (*m/z*) of isoleucine precursor and quantified product masses were 132 and 86, respectively. A collision energy of 10 V was used for the transition. The confirmation product mass for isoleucine was *m/z* 69 with a collision energy of 17 V for the transition. The mass to charge ratios (*m/z*) of 13C/ 15N-isoleucine precursor and quantified product masses were 139.2 and 74, respectively. A collision energy of 18 V was used for the transition. The mass to charge ratios (*m/z*) of leucine precursor and quantified product masses were 132 and 86, respectively. A collision energy of 10 V was used for the transition. The confirmation product mass for isoleucine was *m/z* 44 with a collision energy of 22 V for the transition. The mass to charge ratios (*m/z*) of 13C/15N-leucine precursor and quantified product masses were 139.2 and 92, respectively. A collision energy of 18 V was used for the transition. 

### 2.6. Sensory Evaluation

The control formula in this study is commonly used for tube feeding; therefore, it originally does not contain any flavoring agents. In this study, we developed a fortified formula containing fish oil and branch-chained amino acids, which could generate fish odor and bitter taste, respectively. To mask the unpleasant taste and smell, several flavoring agents were added and tested for consumers’ satisfaction. The enriched formula was masked with non-caloric food flavors, i.e., vanilla, strawberry, lemongrass, cooked Japanese rice odor, chocolate, coconut, peanut, almond, coffee, or spearmint. After a preliminary evaluation by two trained panelists, four flavors including vanilla, strawberry, lemongrass, and cooked Japanese rice (Aromatech Flavours Co., Ltd., Bangkok, Thailand) were selected for sensory tests. Eighty healthy volunteers tasted 30 cc each of the control and fortified formulas in random sequence. Then, they rated their liking for color, appearance, odor, taste, mouthfeel, and total acceptability in 5-point hedonic scales (with word anchors, ranging from 5 = “like extremely” to 1 = “dislike extremely” with 3 as “neither like nor dislike”). The average score of at least 3 was considered sensory acceptable. The number of participants giving each score was counted. The percentage of satisfaction was calculated by dividing the number of participants giving scores ≥ 3 by the total number of participants. A previous study recommended that at least 40 participants were required for consumer tests [21]. Therefore, the sample size of 80 used in this study should provide high power and increase the reliability of the data. The sensory test was performed according to a research protocol ethically approved by Mahidol University Central Institutional Review Board, MU-CIRB 2019/171.2506. 

### 2.7. Statistical Analysis

The amounts of EPA and BCAA were quantitated using linear regression of standard curves generated by plotting the ratios of the area under the curves of standards and the respective internal standards. Unpaired t-tests were used to compare the differences in parameters between the fortified and control formulas. Paired t-tests were performed to determine significant differences in the amounts of EPA and BCAA between those of pre-and post-sterilized samples. A comparison of sensory satisfaction score categories between groups was performed by using the Chi-square test. Sensory satisfaction scores between control and fortified formula were compared by using an unpaired t-test. *p*-value < 0.05 was considered statistically significant. All graphing and statistical analyses were performed using GraphPad Prism V.9.0.2 (GraphPad Software, San Diego, CA, USA).

## 3. Results

### 3.1. Microbial Load, Proximate Composition, Nutrients, Osmolality, Color, and pH of the Processed Control and Fortified Formulas

After retort processing, pathogenic microorganisms commonly found in low-acid hermetically sealed food were not detected (Table S2). As shown in Table 2, the energy and macro-nutrient contents of the fortified formula were relatively similar to that of the control formula. However, the fortified formula had a slight reduction in moisture with increases in protein, dietary fiber, and energy. Table 3 shows that after retort sterilization, the average osmolality in the fortified formula (549.80 ± 1.50 mOsm/kg H_2_O) was higher than that of the control formula (428.8 ± 3.304 mOsm/kg H_2_O; *p* < 0.0001). After retort processing, the color of the fortified formula had similar lightness (*p* > 0.99) but was redder (*p* < 0.0001) and yellower (*p* < 0.0001) than the control formula. Both formulas were quite neutral, as the control and fortified formula had pH of 6.70 and 6.59, respectively. Figure S2 shows a comparison of the visual color before and after retort in both formulas. 

### 3.2. Rheological Properties and Texture of the Processed Control and Fortified Formulas

**Figure 1:**
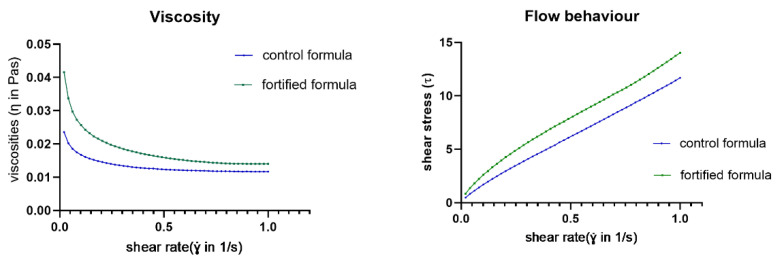
Viscosity and flow curves of retorted control and fortified formulas at different shear rates. The lines represent the corresponding power of increased shear rate.

As shown in Figure 1 and Table 4, the fortified formula had a higher viscosity and shear stress than the control formula. Though the control formula had flow behavior close to the Newtonian pattern, its viscosity was closer to a non-Newtonian (pseudoplastic) pattern. The fortified formula had viscosity and flow behavior in a non-Newtonian (pseudo plastic) pattern. IDDSI flow test showed that all content of both formulas completely flowed through the syringe after 10 s (Figure S3). Thus, both formulas were considered as IDDSI level 0. 

### 3.3. Effect of Retort Sterilization on BCAA

**Figure 2:**
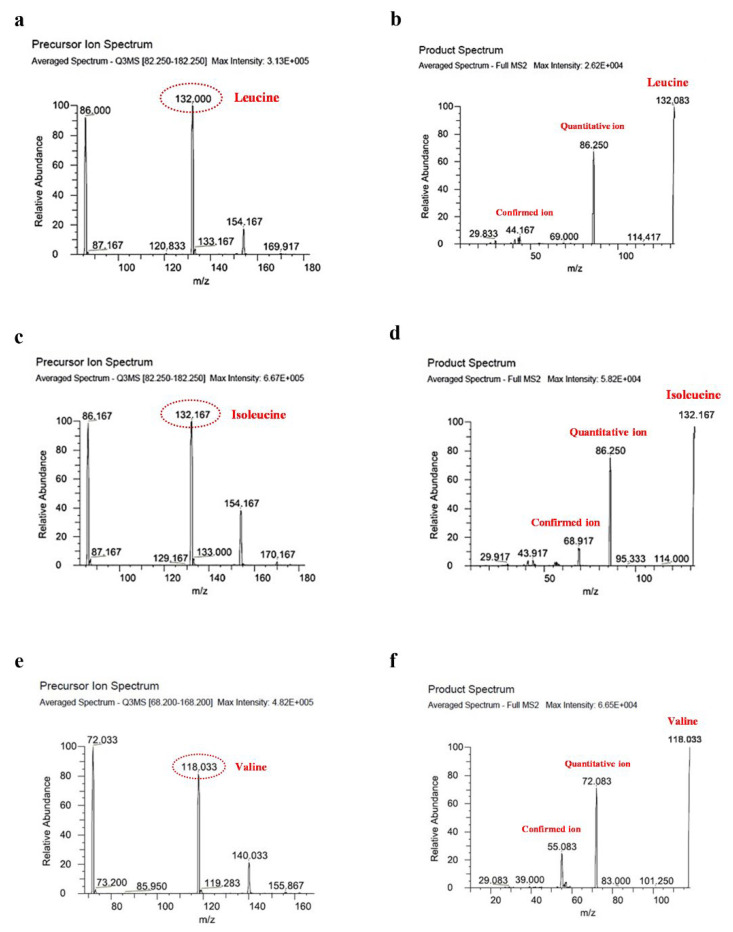
Mass spectra of the precursor and product ion masses of branch-chain amino acids (leucine, isoleucine, and valine), scanned by LC-MS/MS. Precursor ion and product ion spectrums of leucine (**a**,**b**), isoleucine (**c**,**d**), and valine (**e**,**f**), respectively. The number in the oval represents the mass per charge (*m*/*z*) ratio of the precursor. The *m/z* values for quantitative and confirmed product ions for each amino acid are labeled.

**Figure 3:**
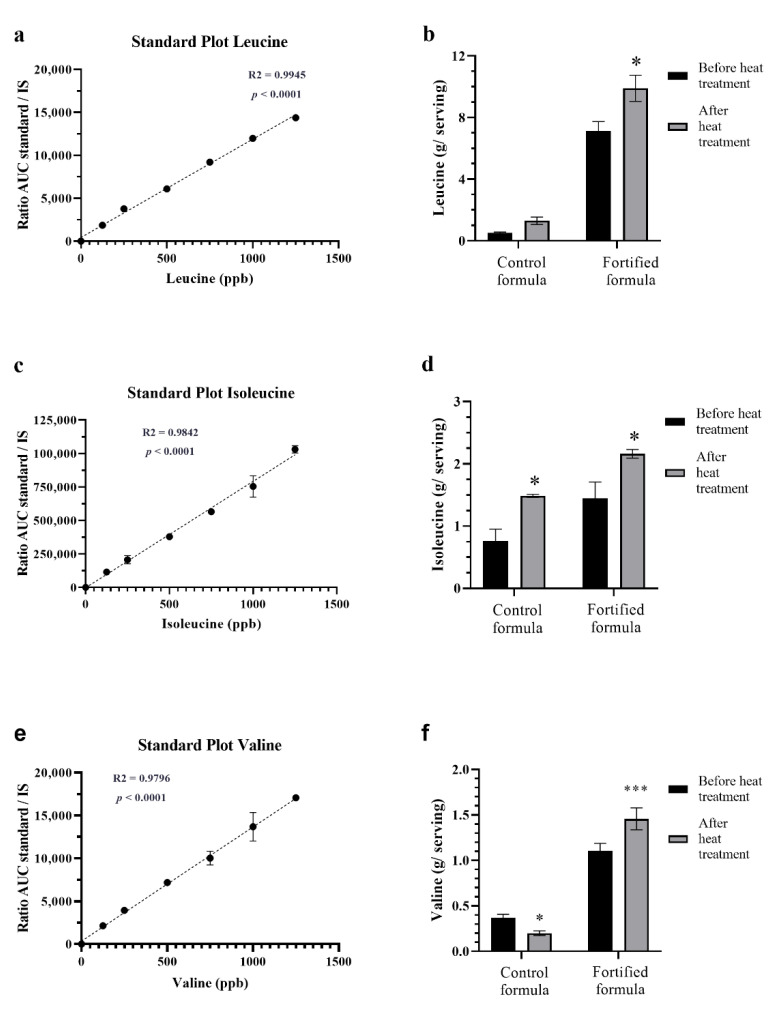
Effect of retort sterilization on the amount of branch-chain amino acids in control and fortified formulas. Standard curves of leucine (**a**), isoleucine (**c**), and valine (**e**), obtained from the ratio of area under the curve (AUC) between respective standards and internal standards and the concentrations of each amino acid, analyzed by LC-MS/ MS. R^2^ and p-values were obtained by using linear regressions. Bar graphs show the amounts of leucine (**b**), isoleucine (**d**), and valine (**f**) in the control and fortified formulas before and after retort processing. * and *** mean *p* < 0.05 and 0.001, comparing before and after retort using paired t-tests.

The chromatograms of BCAA in standard solutions are shown in Figure 2. Mass chromatograms are shown for the BCAA and their stable label internal standards. Selected reaction monitoring transitions were *m/z* 132.167−86.250 for isoleucine; *m/z* 132.083−86.250 for leucine; and *m/z* 118.033−72.083 for valine. Compared with their values before sterilization, there was a significant increase in the amounts of leucine (*p* = 0.0184) isoleucine (*p* = 0.0135), and valine (*p* = 0.0002) in the fortified formula after retort sterilization (Figure 3). For the control formula, the amounts of isoleucine and valine were significantly increased. Leucine in the control formula also increased but not to a significant extent. 

### 3.4. Effect of Retort Sterilization on EPA

**Figure 4:**
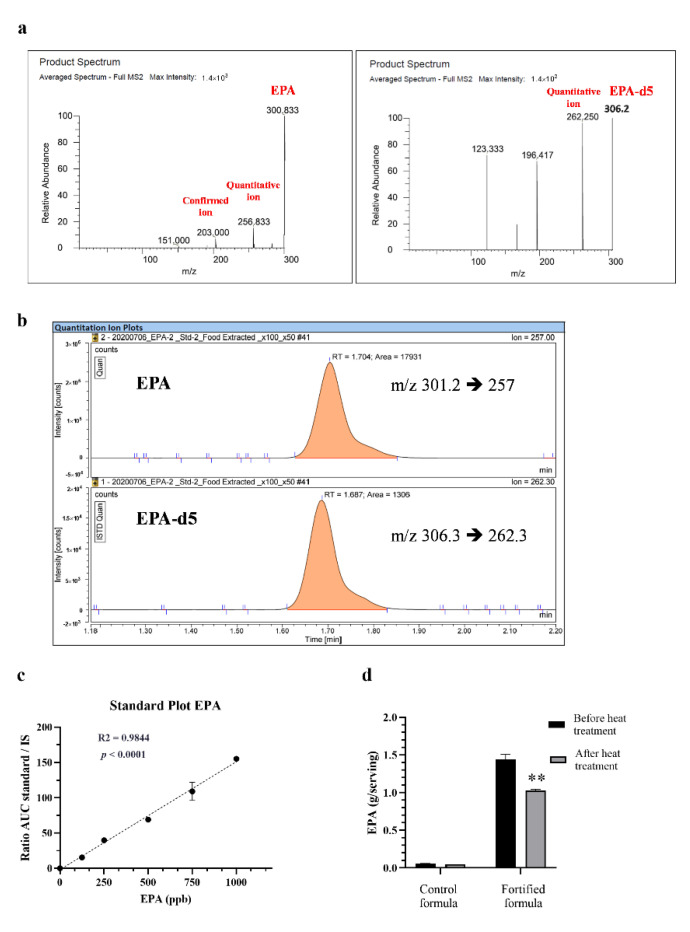
Effect of retort sterilization on the amount of EPA in control and fortified formulas. Precursor ion spectrum and product spectrum of EPA (**a**)-left panel) and the internal standard, EPA-d5 (**a**)-right panel). The number under EPA or EPA-d5 represents the mass per charge (*m*/*z*) ratio of the precursor (b). Standard curves of EPA (**c**), obtained from the ratio of area under the curve (AUC) between respective standards and internal standards and the concentrations of each amino acid, analyzed by LC-MS/ MS. R2 and *p*-values were obtained by using linear regressions. Bar graphs show the amount of EPA (**d**) in the control and fortified formulas before and after retort processing. ** means *p* < 0.01, comparing before and after retort by using paired t-tests.

EPA was not an ingredient in the initial food powder. Measurement of EPA was conducted to identify the quantity of EPA before and after heat treatment with the presence of a complete nutrition food matrix. Compared to the pre-sterilized sample, Figure 4 shows a 30% decrease in EPA in the post-sterilized fortified formula (*p* = 0.0016). 

### 3.5. Sensory Evaluation of Fortified Formula

**Figure 5:**
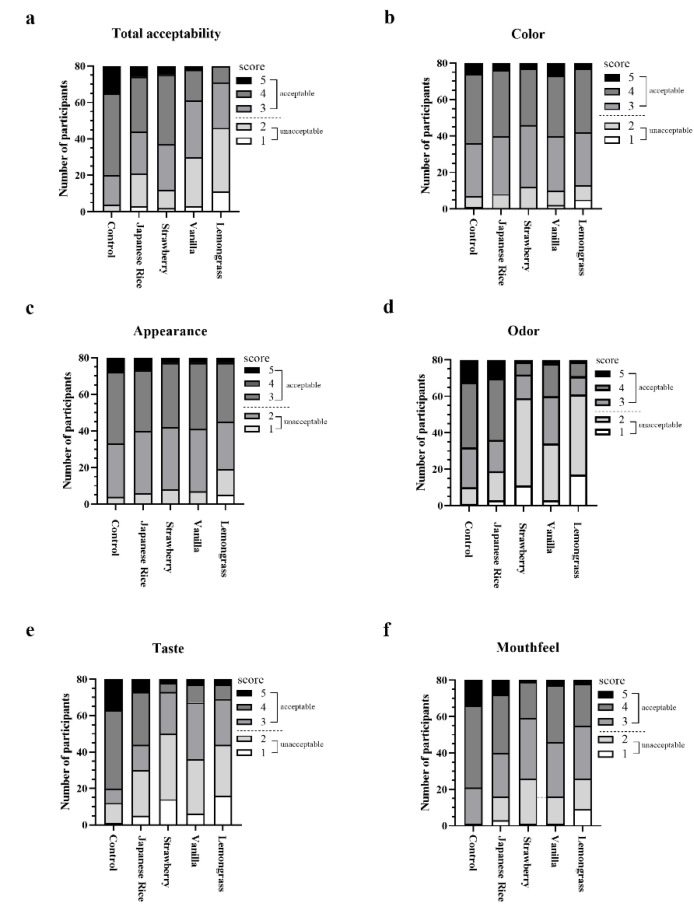
Acceptability of fortified formulas with different flavors. The stacked bars show the number of participants who assigned different scores on 5-point hedonic scales from 1 to 5 for total acceptability (**a**), color (**b**), appearance (c), odor (**d**), taste (**e**), and mouthfeel (**f**) of the fortified formula with Japanese rice, strawberry, vanilla, and lemongrass flavors. A score above 3 is acceptable.

**Figure 6:**
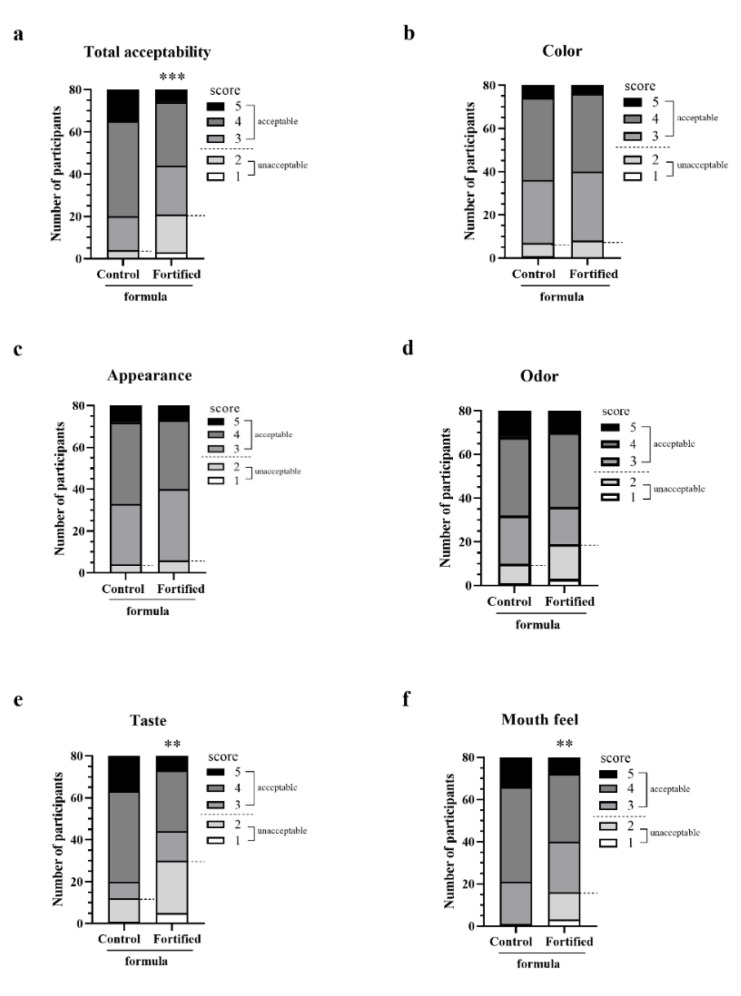
Results of sensory evaluation for control and fortified formulas of the rice-flavored control and fortified formulas. The stacked bars show the number of participants who assigned different scores on 5-point hedonic scales from 1 to 5 for total acceptability (**a**), color (**b**), appearance (**c**), odor (**d**), taste (**e**), and mouthfeel (**f**) of the control and fortified formulas with Japanese rice flavor. A score above 3 is acceptable. (***) and (**) mean *p* < 0.001 and < 0.01, respectively, obtained by chi-square tests.

As shown in Table 5, Japanese rice flavor fortified formula was the only one that received average sensory satisfaction scores above 3 for all parameters. The average sensory satisfaction scores for color, appearance and odor of the Japanese flavor one were not different from those of the control formula. However, the average scores for total acceptability, taste, and mouthfeel were lower than those for the control. Interestingly, the taste, mouthfeel and total acceptability of the Japanese rice flavor fortified formula were significantly better than those of the other flavors. Figure 5 shows that the Japanese rice flavor fortified formula consistently earned the highest proportions of acceptable scores (≥3) from participants. Figure 6b–d shows no difference in the satisfactory score categories for color, appearance, and odor between control and Japanese rice flavored enriched formula. In contrast, the proportions of acceptable scores for taste, mouthfeel, and overall acceptability were significantly higher for the control formula (Figure 6a,e,f). Nevertheless, Figure 6a shows that over 80% of panelists accepted the fortified complete nutrition drink. 

## 4. Discussion

Accurate preparation of nutrition drinks is very critical yet challenging for home caregivers and hospital staff [22,23,24]. Therefore, a ready-to-eat complete nutrition drink is an ideal product choice in both household and health services [22,25]. Although no standard guideline has established for the recommended daily dose of EPA and BCAAs, specialty complete nutrient formulas fortified with amino acids and fatty acids are in increasing demand, especially for cancer patients and elderly people at risk of sarcopenia [12,13,26]. Nevertheless, development of such a product has several concerns including shelf stability, nutrient and functional values, and physical and sensory properties. This study demonstrated that it is feasible to formulate a ready-to-eat complete nutrition formula containing EPA and BCAAs. The findings of this work suggest that the fortified formula had 10 kcal more energy, higher fiber, and less moisture along with increased viscosity and osmolality compared to the control formula. The reason behind the change was likely due to the addition of BCAAs and EPA, which increased the total solids. When we titrated equal volumes of both formulas, the solute could be more concentrated, resulting in increased viscosity, nutrient values and osmolality in the fortified formula. A previous study also observed that osmolality increased by 126–136 mOsm/ 100 mL after fortification with protein hydrolysate in pasteurized human milk [27]. Interestingly, BCAAs contents in the functional liquid nutrition formula were well-preserved after retort sterilization. However, a 30% loss of EPA was found after the heat treatment. The color of the fortified formula after retort sterilization was redder and yellower than that of the control formula. However, the color acceptance of the fortified formula was not significantly different from that of the control formula. The color change was likely from the addition of BCAA and heat treatment. Maillard reaction, the non-enzymatic browning of food consisting of amino acids and reducing sugar, usually occurs when the product undergoes a thermal process, which also affects texture and flavor [28]. The fish odor in the fortified formula was effectively masked with Japanese rice flavoring agents, resulting in no significant difference in odor satisfaction scores between both formulas. Though the average satisfactory score for taste of the Japanese rice flavor-fortified formula was still lower than that of the control, its score was significantly better than those of the other flavors. This finding is consistent with the previous work showing that an artificial flavoring agent with a sweet taste can mask bitter oral medication [29]. Although the overall acceptability score for the fortified formula was lower, the Japanese rice flavored one still received over 80% sensory acceptance. 

In this study, the conventional complete nutrition formula contained some BCAA, and the fortified formula contained an additional amount of BCAA. Interestingly, after retort sterilization at 116 °C for 25 min, the concentrations of isoleucine, leucine, and valine were significantly increased in the fortified formula, while a significant increase in isoleucine was also observed in the control formula. A previous study reported that the concentrations of BCAA-rich supplement Aminoleban^®®^ were stable after heating up to 60 min at a maximum temperature of 80 °C [30]. In contrast, another study found a significant decrease in various free amino acids, including valine in infant formula after autoclaving for 5 min at 105 °C and 5600 kg/m^2^ of pressure. The authors speculated that the Maillard reaction was likely the main cause of BCAA degradation [31]. The discrepancy in the results from various studies may be due to differences in heating temperature and amino acid measurement, i.e., total amino acids in our study and free amino acids in the study by Yeung et al., 2006 [31]. In fact, at 116 °C, protein denaturation is highly likely to occur, and the protein structure would be more vulnerable to acid hydrolysis during the sample preparation, resulting in a higher yield of amino acids [32]. Therefore, it is predicted that when a patient consumes our retorted product, complete gastrointestinal digestion will likely occur since the protein has been denatured and opened for access to the enzyme. Future clinical studies are warranted to confirm this speculation. 

In contrast to the amino acids, there was a significant loss of EPA after retort sterilization. This is consistent with a previous study showing a decrease in polyunsaturated fatty acids (PUFAs) in canned fish after heat treatment [11]. The mechanism behind the EPA loss is likely attributable to the increased lipid oxidation level. High temperature could induce free radical generation, followed by secondary oxidized lipid products. The rate of decreasing PUFA depended on the heating profile [33]. In this study, 30% loss of EPA was found after heat treatment. Therefore, in a subsequent batch production process, an additional 30% of fish oil was added as a raw material, yielding a final concentration of EPA in the fortified formula similar to the pre-sterilization level. 

The fortified formula had higher osmolality (534 mOsm/kg), compared with the control formula (393 mOsm/kg). The osmolality of the fortified formula was higher than the normo-osmotic value (300 and 350 mOsm/kg) and was considered hyperosmolar (>500 mOsm/kg) [34]. The osmolality of complete nutrition liquid was shown to play some role in the complications of enteral feeding, such as feeding intolerance and gastrointestinal complications, i.e., osmotic diarrhea [35]. Nevertheless, a previous study in critical patients found no associations between receiving hyperosmolar nutrition and medication via nasogastric tube, and feeding intolerance, diarrhea, constipation, gastric residual volume, nausea, and vomiting [34]. However, an increased risk of diarrhea was found to be associated with hyperosmolar medication via the naso-duodenal route (OR 138.7 (95% CI: 2.33; 8245)) [33]. Therefore, further evaluation in clinical setting research is needed to monitor this product for possible complications. 

The rheological properties of complete nutrition formulas are especially important for nutrition therapy in patients with dysphagia (swallowing difficulty). Though the fortified formula was slightly more viscous than the control formula, the IDDSI levels of both formulas were similar. Thus, for oral intake, the application of the fortified formula should be similar to that of the control. However, the application for tube feeding may be different. The higher viscosity and shear stress at all shear rates observed in the fortified formula suggest that it may flow more slowly in the tube, potentially requiring longer time to finish tube feeding compared to the control. Future clinical studies are needed to confirm this speculation. 

Sensory properties are critical for patient acceptance of complete nutrition drinks. The EPA-BCAA fortified formula had an unwanted fish odor, bitter taste, and bean flavor. Heat treatment such as retort sterilization could generate more volatile compounds and worsen the sensory properties. Previous studies recommended masking the bitterness or brown flavors of amino acids with almond, hazelnut, chocolate, coffee, caramel, rum raisin, and malt [36]. Fish odor is usually masked by a citrus flavor, such as lime or lemongrass [37]. In this study, among the variety of flavors used, we found that rice flavor could provide the most effective masking performance against the unwanted taste and odor of the EPA-BCAA fortified formula. Since the average sensory satisfaction scores for the odor of the fortified formula with Japanese rice flavor were not significantly different from those of the control, Japanese rice flavor seems to effectively mask the fish odor. The 2-acetyl-1-pyrroline (2AP) is considered to be the signature volatile compound contributing to the rice aroma [38]. Further studies on the mechanism of the odor masking effect by 2AP are worthwhile.

The strength of this study was its method of measuring total amino acids by using acid hydrolysis in sample preparations, which could mimic gastrointestinal digestion when consuming the product. Importantly, the equivalent mass of Leu and Ile was difficult to distinguish with single-step identification [18]. Both amino acids came out at highly similar retention times when run through high-performance liquid chromatography (HPLC) and a single quadrupole mass spectrometer (only precursor molecular mass). With the help of triple quadrupole mass spectrometry, LC-MS/MS could distinguish Leu and Ile based on the differential fragment mass [39]. There were some limitations of this study. Although masking the odor and taste with a flavoring agent could make the EPA-BCAA fortified formula sensory acceptable, its overall acceptability score was still significantly lower than that of the control formula. To completely overcome the challenge, microencapsulation of fish oil using whey protein concentrate, sodium caseinate and glucose syrup were shown to reduce lipid oxidation and, therefore, lessen the fish odor [40]. Future studies are warranted to develop an affordable technology to improve the sensory properties of the fortified nutritious drink. As a last note, the product was processed by retort in order to make it shelf-stable. Since the retort condition achieved F0 = 6 and no detectable pathogenic bacteria, the product could be kept at room temperature for at least 6 months according to Thai Food and Drug Administration standards. A future study to identify the shelf-life of the product at room temperature is warranted.

## 5. Conclusions

The findings suggested that it is feasible to formulate a ready-to-eat shelf-stable complete nutrition drink containing EPA and BCAA. Retort at 116 °C for 25 min was selected for sterilization. Such processing resulted in no loss of BCAA but a 30% loss of EPA. Thus, a 30% addition of fish oil should be supplied as a raw material for production to achieve desirable EPA content in the finished product. Compared to the control formula, the processed fortified formula had slightly higher protein and fiber contents, along with increased osmolality and viscosity. However, its IDDSI texture category did not change. Among the flavors tested, the fortified formula with Japanese rice flavor received the highest satisfaction scores, with over 80% sensory acceptance. The fish odor was effectively masked with a Japanese rice flavoring agent, while the bitter taste was reduced. The present study indicated that retort technology could be an effective sterilization method for special complete nutrition formulas to be used in personalized nutrition. Clinical studies are warranted to investigate its absorption kinetics and functional efficacy. 

## Figures and Tables

**Table 1:** Ingredients of the control and fortified formulas (250 g/serving).

Ingredient	Control Formula	Fortified Formula
Maltodextrin	22.14	22.14
Sucrose	11.00	11.00
Soy protein	9.37	9.37
Rice bran oil	7.61	7.61
Multimineral mixture	1.25	1.25
Fructo-oligosaccharide (FOS)	1.24	1.24
Sodium caseinate	0.81	0.81
MCT oil	0.66	0.66
Multivitamin mixture	0.19	0.19
EPA	-	1.10
Leucine	-	1.66
Isoleucine	-	0.41
Valine	-	0.41

MCT = medium-chain triacylglycerides; EPA = eicosapentaenoic acid.

**Table 2:** Comparison of energy and macro-nutrients in the control and fortified complete nutrition formulas after retort sterilization.

Parameter	Control Formula	Fortified Formula
Energy (kcal) Moisture (g)	98.74 84.08	108.65 82.16
Carbohydrate (g)	13.93	14.03
Protein (g)	3.60	4.20
Fat (g)	3.18	3.97
Dietary fiber (g)	0.32	0.76
Ash (g)	0.61	0.54

The table shows the mean nutrient amount from duplicates of three random samples.

**Table 3:** Comparison of color, osmolality and pH in the control and fortified complete nutrition formulas after retort sterilization.

Parameter	Control Formula	Fortified Formula	*p*-Value
Osmolality (mOsmol/kg H_2_O)	428.80 ± 3.30	549.80 ± 1.50	<0.0001
Color L *	65.27 ± 0.14	65.60 ± 0.94	>0.9999
a *	6.99 ± 0.09	8.01 ± 0.44	<0.0001
b *	21.24 ± 0.2	27.42 ± 0.33	<0.0001
pH	6.7	6.59	

Data were expressed as mean ± standard deviation (SD) of osmolality and color from 3 packages each. pH was the average of duplicate measurements from pooled sample. L*-a*-b* is the 3-dimensional CIELAB color system. L* = lightness axis; a* = green-red axis; + = redder; b* = blue-yellow axis, + = yellower. *p*-values were obtained from unpaired t-tests.

**Table 4:** Comparison of viscosity and flow behavior in the control and fortified complete nutrition formulas after retort sterilization at different time points.

Parameter		Viscosity (Pa.s)		*p*-Value
		Control Formula	Fortified Formula	
Shear rate (1/s)	100	0.016 ± 0.001	0.024 ± 0.002	<0.0001
	250	0.013 ± 0.001	0.019 ± 0.001	0.0002
	500	0.012 ± 0.001	0.016± 2.567 $\times 10^{-4}$	0.006
	750	0.012 ± 3.719 $\times 10^{-4}$	0.014 ± 6.930 $\times 10^{-4}$	0.042
	1000	0.012 ± 2.758 $\times 10^{-4}$	0.014± 4.950 $\times 10^{-4}$	0.062
**Parameter**		**Flow Behavior (Shear Stress: Pa)**		***p*-Value**
		**Control Formula**	**Fortified Formula**	
Shear rate (1/s)	100	1.60 ± 0.150	2.493 ± 0.173	0.0251
	250	4.038 ± 0.536	4.984 ± 0.191	0.0177
	500	6.102 ± 0.269	7.981± 0.131	0.0001
	750	8.724 ± 0.281	10.758 ± 0.052	<0.0001
	1000	11.501 ± 0.276	14.002 ± 0.049	<0.0001

**Table 5:** Sensory satisfaction scores for the control and various flavor-fortified formulas.

Formula	Total Acceptability	Color	Appearance	Odor	Taste	Mouthfeel
Control	3.89 ± 0.76	3.53 ± 0.80	3.64 ± 0.73	3.61 ± 0.92	3.80 ± 0.97	3.90 ± 0.69
Japaneserice	3.23 ± 1.01 ****	3.45 ± 0.74	3.51 ± 0.76	3.40 ± 1.06	3.10 ± 1.13 ***	3.36 ± 1.00 ***
Strawberry	2.84 ± 0.93 ****	3.45 ± 0.90	3.43 ± 0.78	2.89 ± 1.04 ****	2.56 ± 1.07 ****	3.09 ± 0.93 ****
Vanilla	2.85 ± 0.89 ****	3.44 ± 0.88	3.44 ± 0.71	2.81 ± 0.92 ****	2.68 ± 0.92 ****	3.25 ± 0.85 ****
Lemongrass	2.58 ± 0.88 ****	3.31 ± 0.77	3.41 ± 0.72	2.24 ± 0.85 ****	2.31 ± 0.92 ****	2.94 ± 0.82 ****

Data are expressed as mean ± standard deviation (SD) of the average sensory scores. (****) and (***) represent *p* < 0.0001 and 0.001, respectively, compared to control formula. *p*-values were obtained using ANOVA followed by Bonferroni’s multiple comparison test.

## Data Availability

The data presented in this study are available upon reasonable request to the corresponding author.

## Supplementary Materials

The following supporting information can be downloaded at: https://www.mdpi.com/article/10.3390/foods11142023/s1, Figure S1: The graphical plot of heat penetration study of the retort sterilization process; Figure S2: Color of the control and fortified complete nutrition drink before and after retort sterilization. Figure S3: Texture categorization of complete and fortified formulas according to IDDSI. Table S1: Selected parent-product ion m/z transitions and MS conditions used for BCAA and EPA quantification by LC-MS/MS. Table S2: Microbial assessment of the control and fortified formulas.
