# Supplementary material for: Loss of Eicosapentaenoic Acid (EPA) after Retort Sterilization of the EPA-BCAA Fortified Complete Nutrition Drink
Source: Foods. 2022 Jul 8;11(14):2023. doi: 10.3390/foods11142023 (PMC9320311; doi:10.3390/foods11142023)
Supplement: Supplementary file 1 [file foods-11-02023-s001.zip › foods-1778818-supplementary.pdf]

Figure S1: The graphical plot of heat penetration study of the retort sterilization process

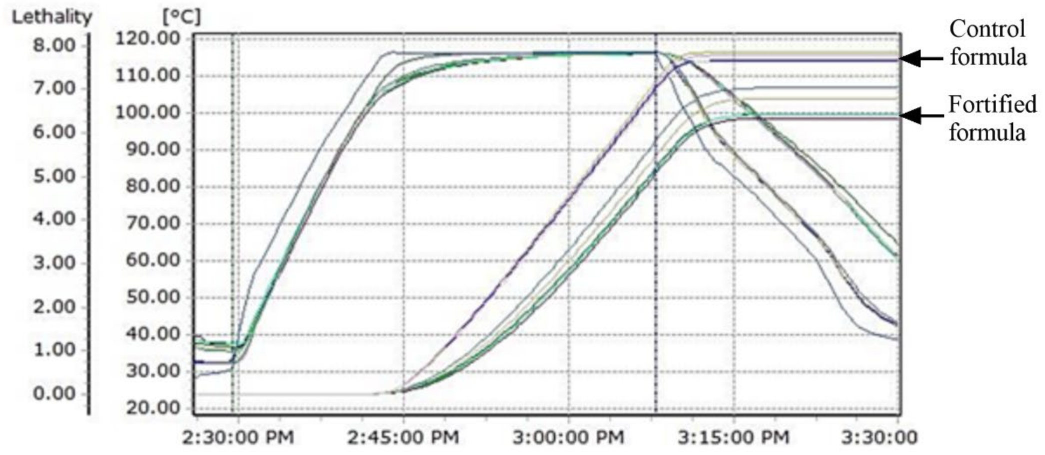

Test run 2 : Process 116°C / 25 minutes

	Name	Description	Start Time	End Time	Max. Value
	LC 01 (F0) Lethality 1	L-Calculation	2:00:00 PM	3:30:00 PM	7.65
	LC 01 (F0) Lethality 2	L-Calculation	2:00:00 PM	3:08:00 PM	7.03
	LC 02 (F0) Lethality 1	L-Calculation	2:00:00 PM	3:30:00 PM	6.44
	LC 02 (F0) Lethality 2	L-Calculation	2:00:00 PM	3:08:00 PM	5.21
	LC 03 (F0) Lethality 1	L-Calculation	2:00:00 PM	3:30:00 PM	6.34
	LC 03 (F0) Lethality 2	L-Calculation	2:00:00 PM	3:08:00 PM	5.08
	LC 04 (F0) Lethality 1	L-Calculation	2:00:00 PM	3:30:00 PM	7.04
	LC 04 (F0) Lethality 2	L-Calculation	2:00:00 PM	3:08:00 PM	5.85
	LC 05 (F0) Lethality 1	L-Calculation	2:00:00 PM	3:30:00 PM	6.43
	LC 05 (F0) Lethality 2	L-Calculation	2:00:00 PM	3:08:00 PM	5.16
	LC 06 (F0) Lethality 1	L-Calculation	2:00:00 PM	3:30:00 PM	6.80
	LC 06 (F0) Lethality 2	L-Calculation	2:00:00 PM	3:08:00 PM	5.49
	LC 07 (F0) Lethality 1	L-Calculation	2:00:00 PM	3:30:00 PM	7.85
	LC 07 (F0) Lethality 2	L-Calculation	2:00:00 PM	3:08:00 PM	7.20
	LC 08 (F0) Lethality 1	L-Calculation	2:00:00 PM	3:30:00 PM	7.78
	LC 08 (F0) Lethality 2	L-Calculation	2:00:00 PM	3:08:00 PM	7.10
	LC 09 (F0) Lethality 1	L-Calculation	2:00:00 PM	3:30:00 PM	7.65
	LC 09 (F0) Lethality 2	L-Calculation	2:00:00 PM	3:08:00 PM	6.95

Note: Lethality 1 – Lethality calculation from both heating and cooling (included F-cooling)
Lethality 2 – Delivered lethality from heating cycle only.

The graph shows the heat penetration attributes (retort temperature, core temperature, F0 value) of complete nutrition drink thermally processed by batch-overpressure water spray retort in retort pouch based on temperature (°C) and time (minutes). The mixture was preheated to 65°C before filling, and the initial temperature prior to processing was 35°C. Lethality (F0) of 5.08 and 6.30 for the fortified and control formula were achieved at 68 min and 90 min, respectively.

Table S1. Selected parent-product ion m/z transitions and MS conditions used for BCAA and EPA quantification by LC-MS/MS

Compound	Retention time (min)	Molecular weight	Precursor ion (m/z)	Product ion (m/z)	Collision energy (eV)	RF lens
Leucine	3.01	138.12	132.0	44.16	25V	59
Isoleucine	3.01	138.12	132.16	68.91	17.68V	59
Valine	2.36	123.10	118.03	72.08	10.25V	56
EPA	2.21	302.50	300.83	256.83	14V	99

Table S2. Microbial assessment in the control and fortified complete nutrition formulae after retort sterilization

Parameter	Control formula	Fortified formula
Flat sour mesophile/ml	Not detected	Not detected
Putrefactive anaerobe/ml	Not detected	Not detected
Flat sour thermophile/ml	Not detected	Not detected
Thermophilic anaerobe/ml	Not detected	Not detected

Data was obtained from 7 packages each, following AOAC methods for the low-acid canned food. The tests were performed by ISO/ IEC 17025 certified laboratory.

Figure S2: Color of the control and fortified complete nutrition drink before and after retort sterilization

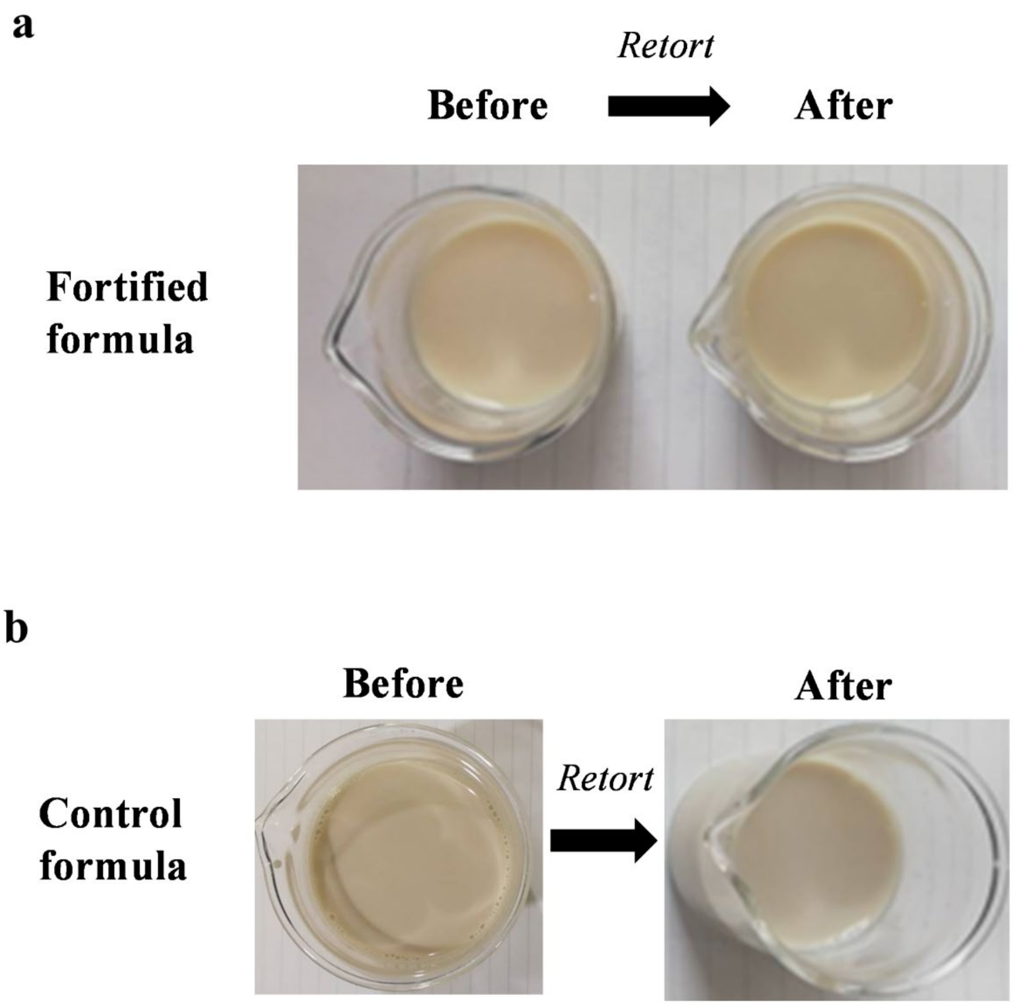

The picture shows the appearance of fortified (**a**) and control (**b**) formulae compared before and after retort processing.

Figure S3: Texture categorization of complete and fortified formulae according to IDDSI

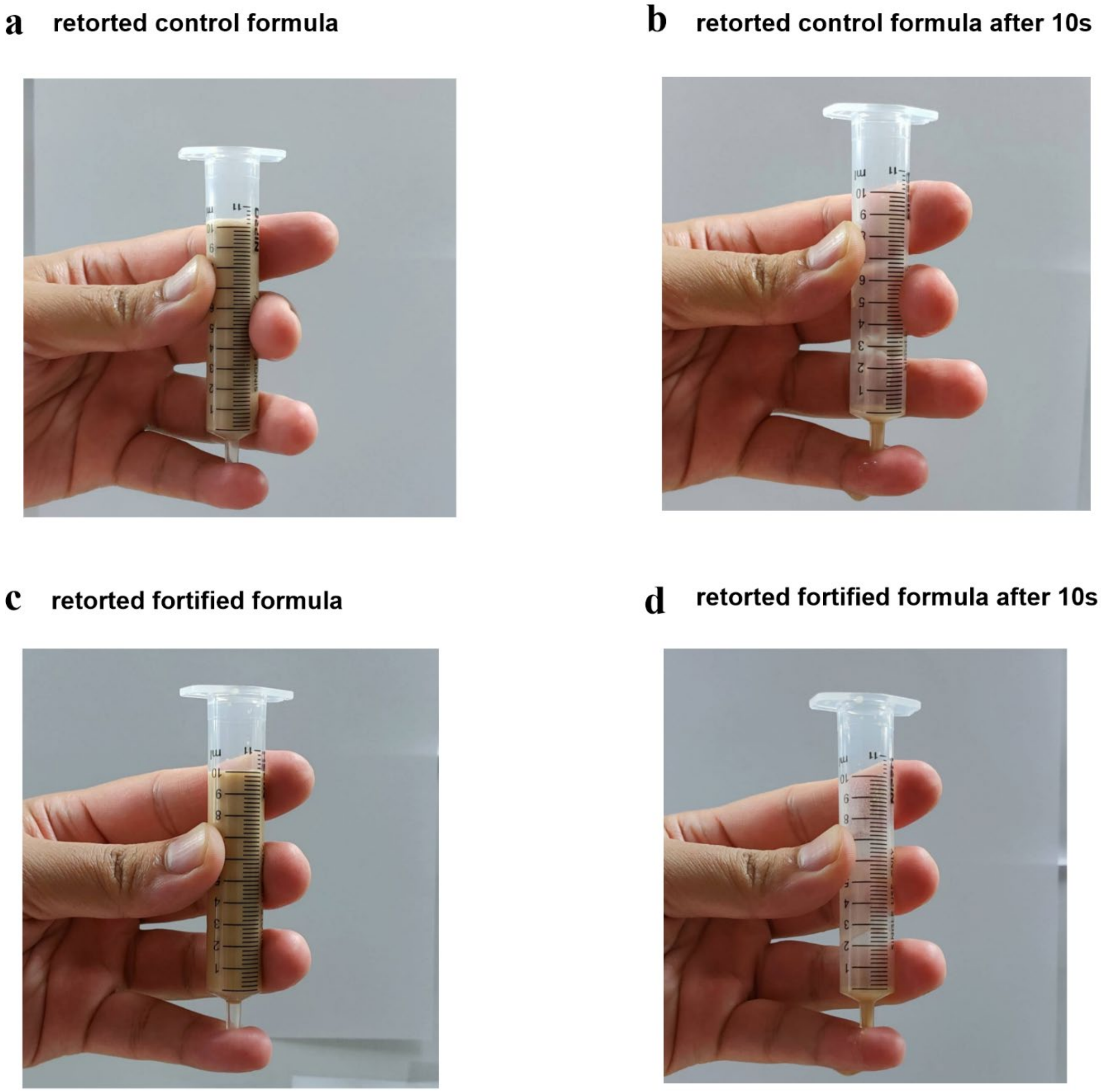

The pictures showed the amount of complete nutrition drink in the syringe before (**a and c**) and after 10 s (**b and d**) of the IDDSI flow test for the control and fortified formula, respectively.
